# The role of attention in processing morphologically complex spoken words: an EEG/MEG study

**DOI:** 10.3389/fnhum.2012.00353

**Published:** 2013-01-09

**Authors:** Alina Leminen, Minna Lehtonen, Miika Leminen, Päivi Nevalainen, Jyrki P. Mäkelä, Teija Kujala

**Affiliations:** ^1^Cognitive Brain Research Unit, Cognitive Science, Institute of Behavioural Sciences, University of HelsinkiHelsinki, Finland; ^2^Department of Psychology and Logopedics, Åbo Akademi UniversityTurku, Finland; ^3^Finnish Centre of Excellence in Interdisciplinary Music ResearchJyväskylä, Finland; ^4^BioMag Laboratory, HUSLAB, Hospital District of Helsinki and UusimaaHelsinki, Finland; ^5^Cicero Learning, University of HelsinkiHelsinki, Finland

**Keywords:** attention, morphology, auditory, inflected, derived, MEG, ERP, lexicon

## Abstract

This study determined to what extent morphological processing of spoken inflected and derived words is attention-independent. To answer these questions EEG and MEG responses were recorded from healthy participants while they were presented with spoken Finnish inflected, derived, and monomorphemic words. In the non-attended task, the participants were instructed to ignore the incoming auditory stimuli and concentrate on the silent cartoon. In the attended task, previously reported by Leminen et al. (2011), the participants were to judge the acceptability of each stimulus. Importantly, EEG and MEG responses were time-locked to the onset of critical information [suffix onset for the complex words and uniqueness point (UP) for the monomorphemic words]. Early after the critical point, word type did not interact with task: in both attended and non-attended tasks, the event-related potentials (ERPs) showed larger negativity to derived than inflected or monomorphemic words ~100 ms after the critical point. MEG source waveforms showed a similar pattern. Later than 100 ms after the critical point, there were no differences between word types in the non-attended task either in the ERP or source modeling data. However, in the attended task inflected words elicited larger responses than other words ~200 ms after the critical point. The results suggest different brain representations for derived and inflected words. The early activation after the critical point was elicited both in the non-attended and attended tasks. As this stage of word recognition was not modulated by attention, it can be concluded to reflect an automatic mapping of incoming acoustic information onto stored representations. In contrast, the later differences between word types in the attended task were not observed in the non-attended task. This indicates that later compositional processes at the (morpho)syntactic-semantic level require focused attention.

## Introduction

In psycholinguistics and cognitive neuroscience of language, numerous studies have attempted to elucidate the issue of whether morphologically complex words such as “work+s” (inflection) and “work+er” (derivation) are accessed as full entities or via their constituent morphemes. Some theoretical models postulate automatic decomposition of all morphologically complex words at the early stages of processing (Taft and Forster, 1975; Meunier and Longtin, 2007; Rastle and Davis, 2008) whereas other models propose that all words are represented and processed in their full-form (Butterworth, 1983). Dual-route models, in turn, claim that both full-form storage and decomposition are possible, depending on the properties of a word, such as word formation type (e.g., inflection vs. derivation), affixal salience, or word frequency (Frauenfelder and Schreuder, 1992; Niemi et al., 1994; Schreuder and Baayen, 1995). Several models propose (at least to some extent) distinct stages of morphological processing: early access to form-based representations, and later activation and integration of semantic and syntactic features of complex words (e.g., Schreuder and Baayen, 1995; Taft, 2004; Meunier and Longtin, 2007). Most studies on the neural correlates of complex words have focused on the visual representation and processing, and the recognition of spoken complex words has received considerably less attention. Moreover, the dependence of morphological processing of spoken words on attention is a matter of little scrutiny. We investigated the extent of automaticity of morphological processing in a task directing focused attention away from the auditory stimuli. The results of this non-attended task are compared to those of the attended task previously reported in Leminen et al. (2011) in which the same participants were to attend to these same stimuli and judge their acceptability.

The role of word formation type in the representation and processing of morphologically complex words has been a matter of controversy. Several behavioral studies have reported differences between the processing of inflected and derived words (Stanners et al., 1979; Laudanna et al., 1992; Schriefers et al., 1992; Feldman, 1994; Niemi et al., 1994; Clahsen et al., 2003; Marslen-Wilson, 2007), whereas other studies reported no differences between the processing of inflections and derivations (Fowler et al., 1985; Raveh and Rueckl, 2000). Using fMRI, Bozic et al. (2009) observed that derivational affixes in English did not selectively activate left-lateralized fronto-temporal areas as compared with inflected words. EEG and MEG studies directly contrasting inflections and derivations have also observed differences in evoked responses [event-related potentials (ERPs) and event-related fields (ERFs)] for inflections and derivations (Leinonen et al., 2008; Leminen et al., 2011). For instance, in an ERP study, Leinonen et al. (2008) reported that derivationally violated stimuli elicited a larger N400 than correct stimuli, whereas inflectionally violated stimuli elicited an anterior negativity effect. Furthermore, in a combined EEG and MEG study, (correct) inflected words elicited ERPs with a larger left-lateralized negativity than derived words (Leminen et al., 2011). The processing of inflected words also activated more strongly and systematically left superior/middle temporal cortices, whereas for derived words, weaker ERP source amplitudes and less systematic source distribution were observed. However, derived words activated right superior temporal areas more strongly than inflected words. Overall, these fMRI and EEG/MEG results suggest that derivational affixes may not trigger decompositional processes in the same way as inflectional affixes (see also Marslen-Wilson, 2007; Marslen-Wilson and Tyler, 2007; Bozic et al., 2009). The different brain activations for derived and inflected words have been suggested to result from the lexicalization and unpredictability of derivational complexity, i.e., their meaning may or may not be compositional (e.g., clear+ly vs. wit+ness) with respect to the meaning of the morpheme constituents (Bozic and Marslen-Wilson, 2010).

In general, electrophysiological studies on correctly inflected words pitted against matched monomorphemic words typically elicit larger ERP/ERF responses than monomorphemic words (Lehtonen et al., 2007; Leinonen et al., 2009; Vartiainen et al., 2009; Leminen et al., 2011). This inflectional processing cost has also been observed behaviorally with healthy participants (e.g., Niemi et al., 1994; Lehtonen and Laine, 2003) and in aphasic patients (Niemi et al., 1994) as longer reaction times and higher error rates for inflected than monomorphemic or derived words. On the other hand, in electrophysiological measures the processing of derived forms has shown a more inconsistent pattern of results, and some studies have reported left anterior negativity ERPs (Palmovic and Maricic, 2008; Bölte et al., 2009), while others observed N400-like ERPs associated with the processing of incorrectly derived words (Janssen et al., 2006; Leinonen et al., 2008; Leminen et al., 2010; Havas et al., 2012).

Most electrophysiological studies on morphological processing have used visual stimuli and tasks requiring overt attention. Nevertheless, some recent studies have investigated the existence of long-term memory traces for affixes using a passive oddball paradigm with mismatch negativity (MMN), without participants' overt attention (Pulvermüller and Shtyrov, 2006). The MMN studies have suggested existence of specific cortical long-term memory circuits for inflectional affixes in English and Finnish as well as morphological word patterns in Arabic (Shtyrov and Pulvermuller, 2002; Shtyrov et al., 2005; Pulvermüller and Shtyrov, 2009; Boudelaa et al., 2010). These representations were activated without focused attention ~150 ms after the onset of relevant morphological information (Shtyrov and Pulvermuller, 2002; Shtyrov et al., 2005; Pulvermüller and Shtyrov, 2009; Boudelaa et al., 2010). Since the MMNs for these studies with morphologically complex stimuli have been elicited at 100–150 ms after the onset of the relevant information, they suggest that the earliest (with respect to the relevant point in time) stages of linguistic processing may be automatic and not depend on attentional demands (Shtyrov et al., 2010). Additionally, attention-independent word processing was demonstrated in an ERP study by Relander et al. (2008). They reported of a semantic priming effect in the N400 in both the active (attention directed to the stimuli) and passive (attention directed to another modality) tasks, albeit smaller in the passive task.

We investigate the effect of attentional modulation on the processing of morphologically complex spoken words, by comparing ERPs and ERFs when attention is directed to visual modality as opposed to focusing on the spoken words. We also investigate automatic activation of morpheme and lexical representations to obtain information of the nature of such representations in different word types. To this end, the same participants performed a task, which required focused attention on the auditory stimuli, and a task, which required ignoring the auditory information (for a similar design, see Relander et al., 2008). In the non-attended task, the participants were distracted from the auditory stimuli by watching a silent movie to be later able to answer questions related to its content. This task was used to avoid selective attention and eliminate different stimulus-related strategies on brain responses. In the attended task, the participants listened to the stimuli and judged whether they were acceptable in Finnish or not. The effects observed in the attended task have been reported in detail elsewhere (Leminen et al., 2011). In contrast to aforementioned studies using the passive oddball paradigm, in which a very small set of repeated stimuli is typically used, we used many non-repeated stimuli, and thus tapped on more natural non-attended auditory processing. Moreover, we time-locked the stimuli to the point in time where the auditory information crucial for morphological processing was available, i.e., the precise suffix onset for the complex words and uniqueness point (UP) for monomorphemic words, enabling the comparison of the ERP/MEG responses directly starting from the suffix onset. It has been suggested that morphophonological decomposition process segments quickly and automatically all potential inflected, and possibly, also derived forms, into stems and affixes, triggered by their surface phonological properties (Marslen-Wilson and Tyler, 2007). Thus, if morphological decomposition processes, such as morpheme segmentation and integration, are automatic, they should be activated also when stimuli are not attended to. Inflected words elicited larger ERP and MEG responses than monomorphemic and derived words in the attended task (Leminen et al., 2011), suggesting morphological decomposition for inflections. Morphological decomposition might takes place also in the non-attended task, reflected by similar ERP/MEG effects as in the attended task. If, however, some of the morphological processing stages are attention-dependent, differences to the attended task should appear in at least some time windows of ERP/MEG responses.

## Methods

### Participants

Ten healthy right-handed adults (6 males, age range 18–34 years, mean 26 years) participated in the study. All were native speakers of Finnish. None of them reported any hearing defects, linguistic dysfunctions, or neurological disorders. They gave written informed consent to participate in the experiments, which were performed in accordance with the Declaration of Helsinki. Ethical permission for the experiment was issued by the Research Ethics Committee of the Helsinki University Central Hospital.

### Stimuli

The experiment consisted of attended and non-attended tasks [the details of attended task are reported elsewhere (Leminen et al., 2011)]. Four hundred and fifty words were used in each task (900 different stimuli altogether), with 75 in each stimulus condition [150 stimuli in both attended and non-attended task, values for attended task are reported in Leminen et al. (2011)]. One word list consisted of monomorphemic Finnish nouns (e.g., “morsian”/“bride”), another of case-inflected words (including genitive, partitive, essive, and different locatives, e.g., “talo+ssa”/“in a house”), and the third of derived words (including collective, possessive, and caritive suffixes, e.g., “karva+ton”/“hairless”). These inflectional and derivational suffixes have been previously used in several studies of Finnish language (Laine, 1996; Bertram et al., 1999; Vannest et al., 2002; Lehtonen and Laine, 2003; Lehtonen et al., 2007; Leinonen et al., 2009; Leminen et al., 2011). All suffixes were attached to nominal stems. In each inflected or derived word, the base morpheme was different, and there were no repeated stems. Most stems (90%) were morphophonologically transparent, i.e., they did not undergo consonant gradation during insertion of a derivational or inflectional ending.

In order to balance the number of correct and incorrect responses, the study included 225 (altogether 450 in both attended and non-attended tasks) pseudowords containing derivational and inflectional violations, and monomorphemic pseudowords. The violations consisted of items that had an existing noun stem and an existing suffix but their combination was morphophonologically illegal (e.g., kylpy+n, lintu+sto; the correct form being “kylvyn”/“linnusto” with a consonant change in the stem). Monomorphemic pseudowords complied with the phonotactic rules of Finnish (e.g., vorsilo) and they were formed by changing one to three phonemes from an existing monomorphemic Finnish word.

The derived words and inflectional stems were selected using the Finnish corpus (approximately 109 million tokens) composed by the Research Institute for the Languages of Finland, the Finnish IT center for science and the Department of General Linguistics, University of Helsinki. The per million frequency counts were log-transformed. In the non-attended condition, mean log base frequencies for the inflected words were 0.99 per million, for derived items 1.49 per million, and for monomorphemic words 0.12 per million. The log surface frequencies for the inflected, derived, and monomorphemic words were −0.5, −0.51, and 0.82 per million in the non-attended situation, respectively^1^. Stimuli were spoken at a normal rate in a randomized order by a female native speaker of Finnish and recorded directly onto a computer hard-drive, using a 44.1 kHz sampling frequency and 16-bit quantization. The sound level of individual waveforms was group-normalized based on peak RMS value in Adobe Audition version 3.0. Mean stimulus duration for inflected words was 727 ms (*SD* = 81 ms), for derived words 781 ms (*SD* = 71 ms), and for monomorphemic words it was 744 ms (*SD* = 91 ms). Due to variations in the duration of the base morpheme, we were not able to match precisely the whole word durations (all *p*-values < 0.05). However, time-locking of the responses to the precise onset of the critical point ensured that the overall duration of the stimulus was not relevant, because the onsets of the suffixes were matched.

A temporal display of each auditory stimulus was used to establish the time point at which the critical point was presented. For each affixed word, the time point of the suffix onset in each auditory file was marked with a trigger code for time-locking ERPs to critical points. For monomorphemic words, in which there are no suffixes, we set a trigger at the UP. The UP (i.e., the phoneme at which a word deviates from all words that share the same phonemes up to and including the phoneme preceding the UP) was determined by a corpus search. In all words the meaning of the base morpheme (and in the case of monomorphemic words, the whole word) was accessed at the critical point. The mean suffix onset for the inflected words was at 520 ms (*SD* = 92) and for the derived words at 490 ms (*SD* = 69) from stimulus onset. For monomorphemic words, the mean onset of the UP was at 528 ms (*SD* = 77)^2^. We verified that the word endings after the critical point did not acoustically differ in different tasks as reflected by N1 and P2 amplitudes. In this control study, naive participants (not included in the actual study) were passively presented with only the target endings of the base morphemes. The ERP N1 or P2 deflections elicited by inflectional and derivational suffixes did not differ significantly [(*F*_(1, 4)_ = 1.61, *p* = 0.273) and (*F*_(1, 4)_ = 2.24, *p* = 0.209), respectively]. These results verify that the possible differences in morphological processing are not due to acoustic differences between the conditions (see also Leminen et al., 2011).

### Procedure

The experiment consisted in total of 900 stimuli, with attended [results reported in detail in Leminen et al. (2011)] and non-attended (results reported here) tasks containing 450 stimuli, respectively, thus no stimulus was repeated. The stimuli for each task were randomly selected, separately for every participant from the 900 stimuli. As the same participants underwent both attended and non-attended tasks, the order of the tasks was counterbalanced across participants. There were three blocks in each task and the order of blocks was randomized for each participant. Also the words in each block were randomized. The stimuli were presented binaurally through plastic tubes at a comfortable sound level. The inter-stimulus interval was 1500 ms. The stimulus presentation was commanded by a script written in Presentation 12.2 (Neurobehavioral Systems, Albany, USA). Each task lasted approximately 30 min, and the subjects were allowed to take a break between each block and between tasks. In the non-attended task, the participants were asked to watch a silent cartoon without subtitles and to ignore the stimuli presented through headphones and learn the details of the cartoon to complete a subsequent written test. The questionnaire had three questions concerning the events of the film. Each question had three response alternatives.

### Data acquisition

The recordings were performed in an electrically and magnetically shielded room (ETS-Lindgren Euroshield, Eura, Finland) with a Vectorview™ whole head MEG system (Elekta Neuromag®, Elekta Oy, Helsinki, Finland). The dewar was in the seated position. The 306-channel helmet-shaped system consists of 102 sensor elements each comprising two orthogonal planar gradiometers and one magnetometer. The EEG was concurrently recorded with a 60-channel electrode cap using an amplifier integrated in the MEG equipment designed and built for simultaneous EEG and MEG recordings (Virtanen et al., 1996). Additional electrodes were placed on the left and right mastoids. Horizontal EOG was monitored with electrodes placed at the temples and the vertical EOG with electrodes attached above and below the left eye. The reference electrode was attached to the nose and ground electrode to the cheek. The head position inside the recording device was determined by activating four indicator coils, whose position on the head was digitized relative to the preaurical points (*x*-axis: positive to the right) and the nasion (*y*-axis: perpendicular to the *x*-axis, positive toward the nasion; *z*-axis: perpendicular to the *x*–*y* plane, positive superiorly), with an Isotrack 3D-digitizer (Polhemus, Colchester, VT, USA). The EEG and MEG signals were band-pass filtered between 0.1 and 200 Hz and digitized at 600 Hz.

### Data analysis

The continuous MEG raw data were pre-processed off-line using a Spatiotemporal Signal Space Separation method (tSSS) of the MaxFilter™ software (Elekta Neuromag®, Elekta Oy, Helsinki, Finland), to minimize the effects of external interference (e.g., line frequency noise) and artifacts produced by nearby sources (e.g., the heart and dental braces) (Taulu and Simola, 2006). tSSS was performed in a 4-s time window (thus, suppressing frequencies below 0.25 Hz) with the default correlation limit of 0.98. Thereafter, the EEG and MEG data were processed with BESA Research 5.3 Software (BESA GmbH, Munich, Germany). The data were low-pass filtered at 45 Hz. The EEG data were re-referenced off-line to the average of mastoids. Any channels with technical malfunction were replaced by interpolating the data of the surrounding electrode sites (Perrin et al., 1989; Bendixen et al., 2008). The data were further processed by an automatic eye-blink correction using principal component analysis (PCA; Ille et al., 2002), and other remaining artifacts were removed automatically using ±100 μV rejection level for EEG data as well as 1200 fT/cm and 2000 fT rejection level for gradiometers and magnetometers, respectively. Thereafter the EEG and MEG responses were epoched (time-locked in separate averages to both stimulus onset and critical point) and baseline corrected. Data time-locked to the stimulus onset were epoched using a time window of −100 to 1200 ms with a baseline correction of −100 to 0 ms, whereas data time-locked to the critical point were epoched using a time window of −100 to 700 ms and baseline correction of −100 to 0 ms before the critical point. On average, in the stimulus onset and critical point time-locked data, 22 and 25% of trials were excluded after artifact correction, respectively.

### Source modeling

In order to directly compare the effect of attentional modulation, the source modeling strategy utilized in the attended task [reported in detail in Leminen et al. (2011)] was applied also for non-attended task. Source locations of the MEG data were first determined using L1 norm minimum current estimates (MCE) in order to obtain an overview of the spatial distribution of the source activity and to compare it with ECD modeling. The MCE were calculated using the MCE module of the Elekta Neuromag® MEG analysis software (Elekta Oy, Helsinki, Finland). Singular value decomposition (SVD) was applied to reduce the influence of noise. To reduce the temporal instability of L1 solutions, source estimations were performed over time intervals of 5 ms. Minimum-norm current estimates were calculated for each participant and stimulus type individually for consecutive time steps. A spherical head model was used for calculating MNCE solutions for each subject individually. The current estimates of each participant were aligned on the 1231 possible source loci of a triangularized gray matter surface of an average brain surface. Data from different participants were overlaid using Neuromag's standard coordinate system.

After MCE modeling, cortical sources of the magnetic fields were modeled as ECDs for the activity after the critical point. All 204 gradiometers were used in dipole modeling (BESA Research 5.3, 4 shell ellipsoidal head model). To compare the source activity in the attended and non-attended tasks, the same dipole modeling procedure was used in the non-attended task (reported here) as in the attended one (Leminen et al., 2011). First, PCA was applied on the grand average MEG data. PCA demonstrated that in the non-attended task the majority of the total variance (94%) in all word types could be optimally explained by maximum of two mono- or bilateral source patterns [in the attended task reported in Leminen et al. (2011), 95.3% of the total variance was also explainable by two bilateral sources]. In order to directly compare the sources in the non-attended vs. attended tasks, the dipoles were fitted in two time windows (80–120 and 170–210 ms after the critical point) according to the magnetic flux distribution in the attended task (Leminen et al., 2011). The dipoles were fitted to explain as much variance of the whole fit interval as possible. Thus, we used the same fit intervals for both tasks and the same fitting strategy, but the model was fit independently for the two tasks. The quality of the MEG data also allowed a different fit model for every stimulus type.

The activity in the 80–120 ms time window (Source pattern 1) was modeled by one bilateral source (two dipoles, one in each hemisphere) and in the 170–210 ms time window, a second bilateral source (two dipoles, one in each hemisphere) (Source pattern 2) was added to the model (also suggested by PCA). The model with two bilateral sources (4 dipoles altogether) was then applied to the individual data for the two time windows and three word types. Originally, in the attended task, the individual dipoles with poor goodness of fit (GoF) were not included in the final model. Therefore, in order to directly compare the attended and non-attended tasks, the same participants were included in the final solutions, despite this resulting in a rather small sample size (Source pattern 1: eight participants; Source pattern 2: left hemisphere: seven participants and the right hemisphere: six participants). In the 80–120 ms time window, mean GoF was 84% (*SD* = 10) and in the 170–210 ms time window, mean GoF was 73% (*SD* = 13). As the source activity was in general reduced in the non-attended task (Figure 4), this might have affected the GoF of the source activity (in the attended task mean GoF in the attended task for Source pattern 2 was 81%, Leminen et al., 2011).

Furthermore, in order to acquire an overview of the source activity of the EEG data and to compare it with that of the MEG data, we performed distributed source analysis (LORETA) on grand average ERPs in the 80–120, 170–210, and 190–230 ms time windows after the critical point [the latter activation reported previously in Leminen et al. (2011)] using BESA Research 5.3 software.

### Statistical analyses

#### ERP data

Repeated measures ANOVA were conducted on twelve EEG electrodes (F7, F3, F4, F8, T7, C3, C4, T8, P7, P3, P4, P8). The electrodes were divided into three regions of interest (ROI): anterior (F7, F3, F4, F8), central (T7, C3, C4, T8), and posterior (P7, P3, P4, P8). To test hemispheric differences, the electrodes were divided into four additional ROIs: left (F7, T7, P7), left midline (F3, C3, P3), right midline (F4, C4, P4), and right (F8, T8, P8). The mean amplitudes for the stimulus onset time-locked data were calculated in the 700–780 ms time window. This time window was selected on the basis of direct comparison with the results of the ERPs in the attended task (Leminen et al., 2011). For the ERPs time-locked to the critical point, the mean amplitudes were calculated in separate ANOVAs the 80–120, 170–210, and 190–230 ms time windows, again in accordance with the attended task (Leminen et al., 2011). The three ANOVAs for the different time windows contained the mean amplitudes from the attended task [published previously in Leminen et al. (2011)] and the non-attended task (presented here). For each time window of interest, the mean amplitudes were analyzed with Four-Way ANOVA with within-subject factors Task (attended, non-attended), Word Type (monomorphemic, derived, inflected), Anterior-Posterior Axis (anterior, central, posterior), and Laterality (left, left midline, right midline, right). *Post hoc* tests were performed using the least significant differences (LSD) test. All *p*-values were Greenhouse–Geisser corrected for non-sphericity when appropriate.

#### MEG data

The dipole strength differences between conditions were assessed in the 80–120, 170–210, and 190–230 ms time windows after the critical point. The 190–230 ms time window was selected in order to compare the source strengths with the ERP results. The mean source amplitudes in each time window and hemisphere were assessed using three separate repeated measures ANOVAs with factors Task and Word Type (monomorphemic, derived, inflected).

## Results

### Behavioral results

After the experiment, the participants were asked to fill in a questionnaire testing how well they had learned the contents of the film. The performance in this test was highly accurate (mean = 93%, *SD* = 13%).

### ERP results

**Figure 1:**
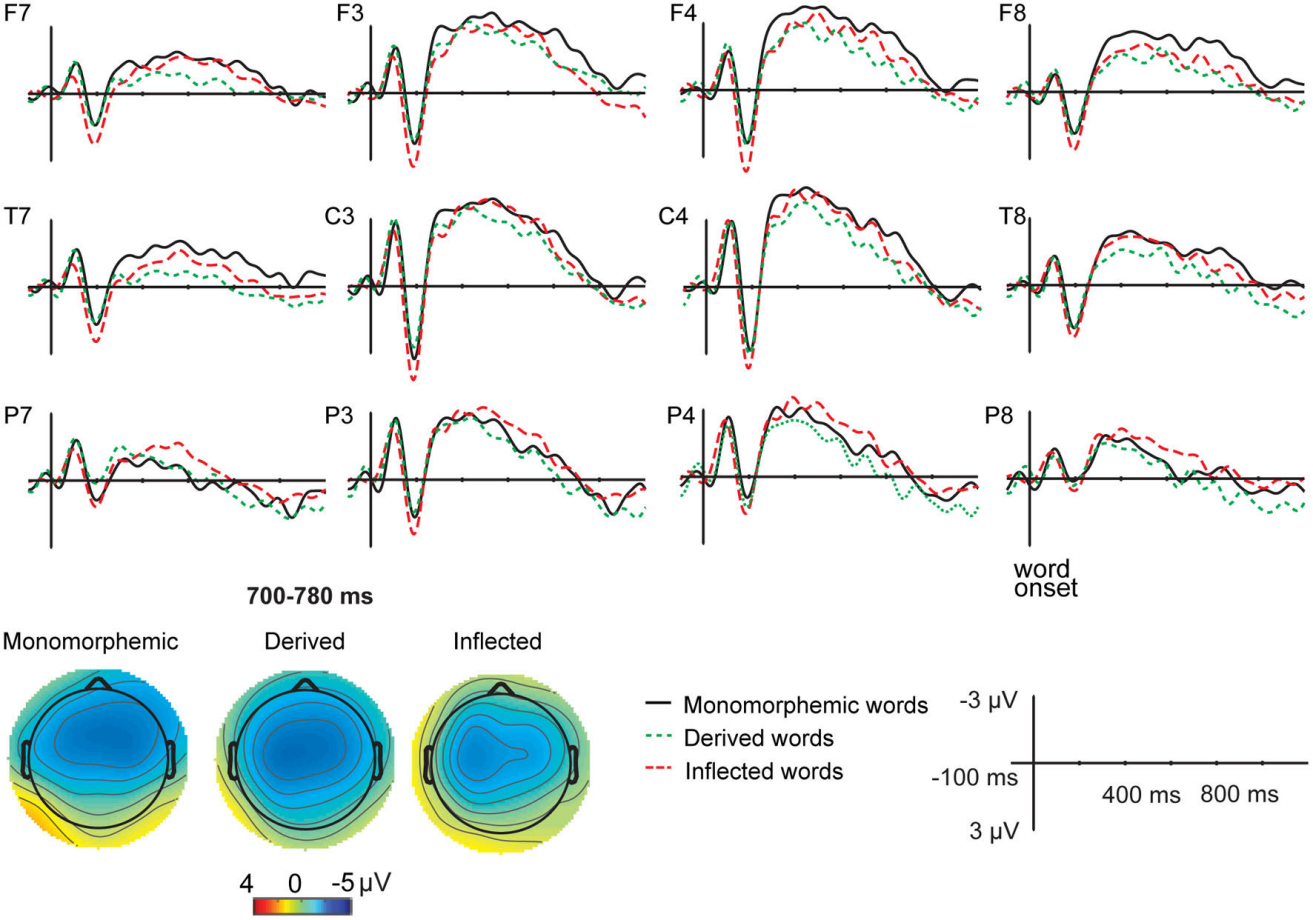
**Grand average ERPs from 12 lateral scalp sites (F7, F3, F4, F8, T7, C3, C4, T8, P7, P3, P4, P8) to monomorphemic words (black solid line), derived words (green dotted line), and inflected words (red dashed line) for the non-attended task data**. Baseline is corrected in the −100 to 0 ms prestimulus interval. Time 0 is the onset of the stimuli. Negative polarity is plotted upwards. The data was low-pass filtered to 20 Hz for illustrating purposes. *X*-axis represents time (milliseconds), *Y*-axis depicts voltage (microvolts, μV). Topographic maps of the distribution of the negativity in the 700–780 ms time window after stimulus onset for monomorphemic, derived, and inflected words.

**Figure 2:**
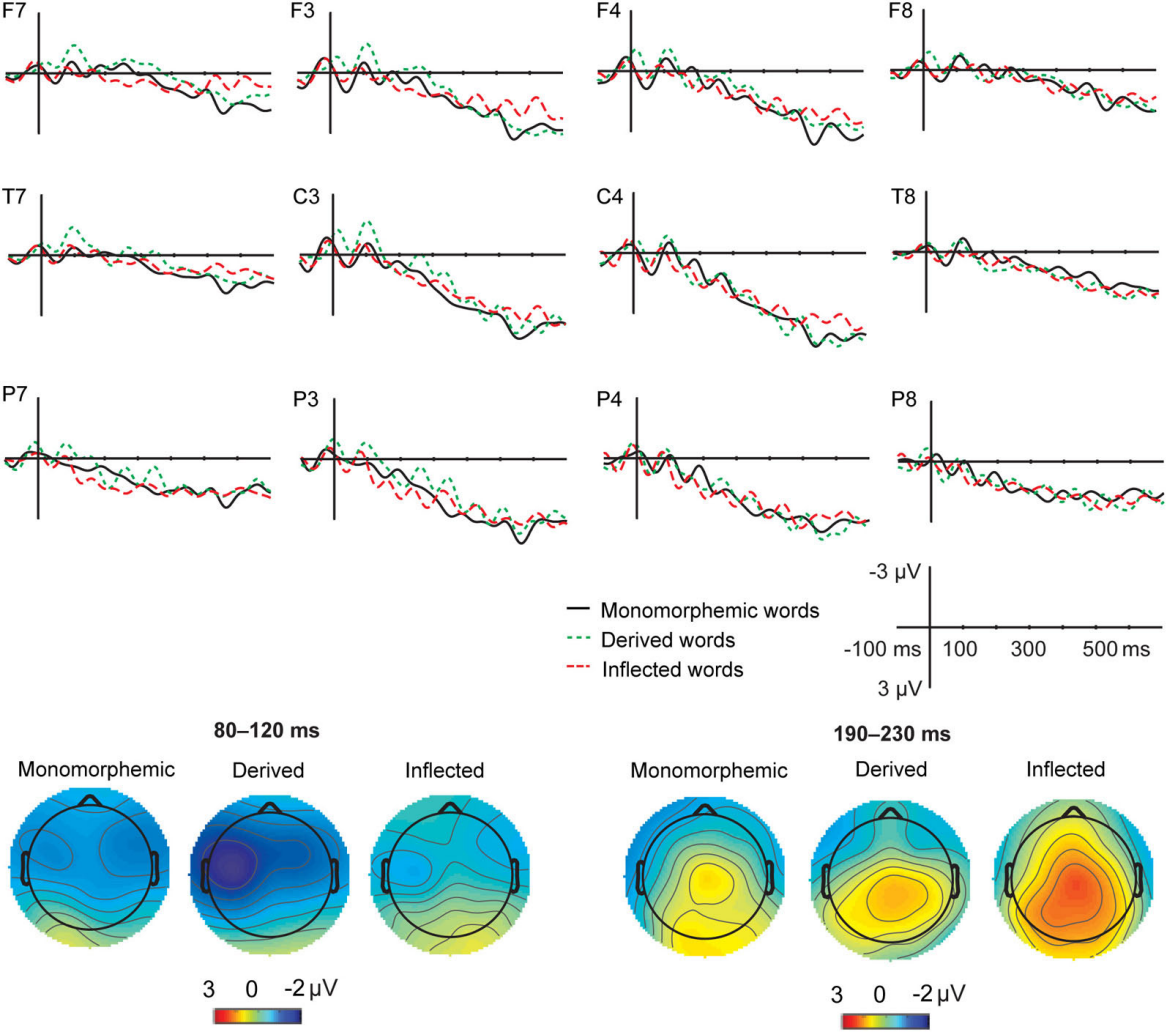
**Grand average ERPs from 12 lateral scalp sites (F7, F3, F4, F8, T7, C3, C4, T8, P7, P3, P4, P8) to monomorphemic words (black solid line), derived words (green dotted line), and inflected words (red dashed line), for the non-attended task data, with baseline correction in the −100 to 0 ms time window before the critical point (i.e., uniqueness point for monomorphemic words; suffix onset for affixed words)**. Time 0 is the onset of the critical point. The data was low-pass filtered to 20 Hz for illustrating purposes. Scales as in Figure 1. Topographic maps of the distribution of the negativity in the 80–120 and 190–230 ms time windows after the critical point for the monomorphemic, derived, and inflected words.

**Figure 3:**
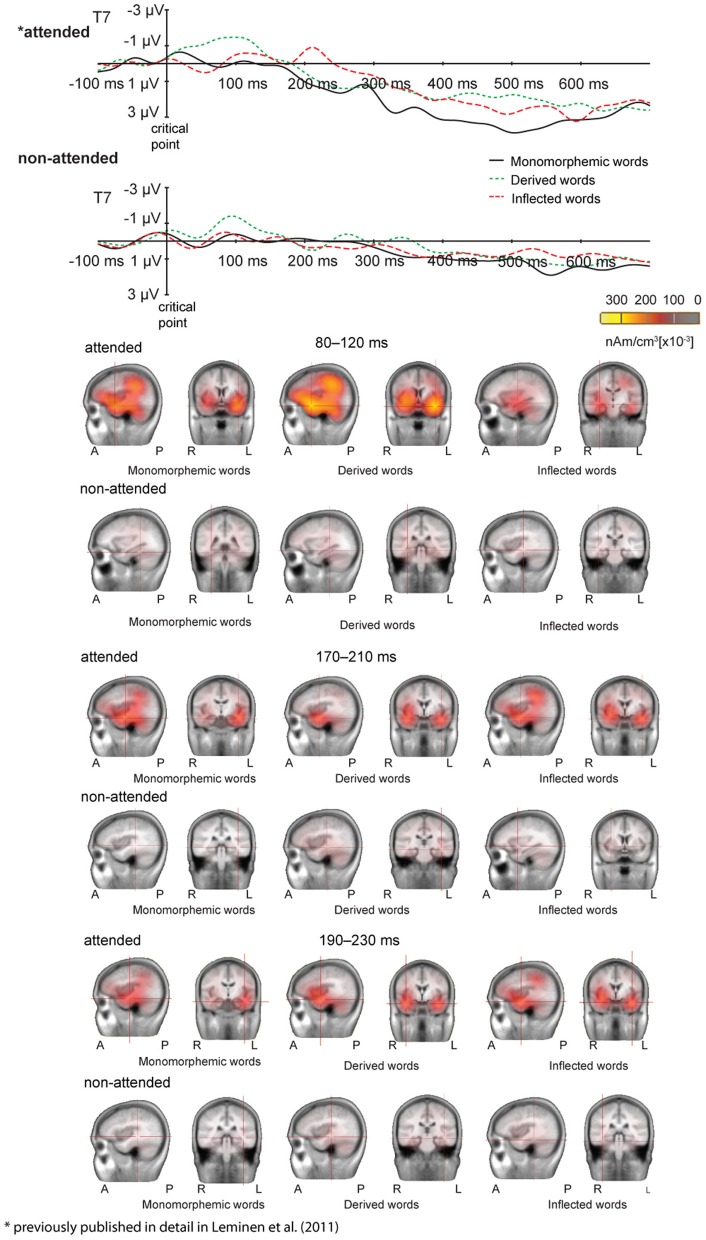
**(above) Grand average ERPs from one representative electrode T7 in the attended and non-attended tasks to monomorphemic words (black solid line), derived words (green dotted line), and inflected words (red dashed line)**. Baseline is corrected in the −100 to 0 ms prestimulus interval. Time 0 is the onset of the critical point. Scales as in Figure 1. (below) LORETA images for grand average ERP responses in the 80–120, 170–210, and 190–230 ms time window after the critical point for monomorphemic, derived, and inflected words in the attended and non-attended tasks. Decreasing the scale resulted in vast increase of activity in the attended task, hence the scale was set to optimally depict activation in both tasks.

Figures 1 and 2 illustrate the grand average ERPs and their scalp distributions in the non-attended task for monomorphemic, derived, and inflected words time-locked to the stimulus onset and to the critical point, respectively. Figure 3 shows ERPs time-locked to the critical point in the attended and non-attended tasks from one representative (T7) electrode. The stimulus onset time-locked ERPs displayed an N400-like negativity for all words. However, no clearly larger negativity for inflected words as compared to other words were observed, unlike in the attended task. In the non-attended task, the derived words elicited larger ERP negativity approximately at 100 ms after the critical point than to the other words. The LORETA analysis on ERP data in attended and non-attended tasks in the 80–120, 170–210, and 190–230 ms time windows after the critical point demonstrates activation in the temporal areas for all word types. Source activation shows stronger activity in the attended than in non-attended tasks in all time windows.

For the stimulus onset time-locked ERPs^3^, in the 700–780 ms time window, Task interacted significantly with Word Type [*F*_(2, 18)_ = 5.52, *p* = 0.031]. *Post hoc* tests showed that in the non-attended task, monomorphemic words elicited a significantly larger negativity than derived words (*p* = 0.038) and inflected words elicited a marginally significant larger negativity than derived words (*p* = 0.097). There were no differences between inflected and monomorphemic words (*p* = 0.516). In the attended task, inflected words elicited a larger negativity than derived words (*p* = 0.030) and monomorphemic words (*p* = 0.033), whereas there were no differences between derived and monomorphemic words (*p* = 0.122).

For the critical point time-locked ERPs, in the 80–120 ms time window, there was no significant main effect of Task (*F* < 1) or significant Task × Word Type interaction (*F* < 1). Task interacted significantly with Anterior-Posterior Axis [*F*_(2, 18)_ = 15.16, *p* < 0.001]. According to *post hoc* tests, in the non-attended task, the amplitudes were more negative at the frontal than at the posterior sites (*p* = 0.002) and at the central than at the posterior sites (*p* < 0.001) but there were no differences between frontal and central sites (*p* = 0.896). In the attended task, the amplitudes were more negative at the frontal than at the central and posterior sites (*p* = 0.047 and *p* < 0.001, respectively). The amplitudes were also more negative at the central than at the parietal electrodes (*p* < 0.001). There was a significant main effect of Word Type [*F*_(2, 18)_ = 4.81, *p* = 0.021]. *Post hoc* tests showed that derived words elicited a larger negativity than inflected words (*p* = 0.029) but there were only marginally significant differences between derived and monomorphemic words (*p* = 0.075) and no reliable differences between inflected and monomorphemic words (*p* = 0.268). Word Type interacted significantly with Laterality [*F*_(6, 54)_ = 2.4, *p* = 0.040]: in the left hemisphere electrodes, derived words elicited a larger negativity than inflected words (*p* = 0.029) and monomorphemic words (*p* = 0.027).

In the 170–210 ms time window, there was a significant main effect of Task [*F*_(1, 9)_ = 37.27, *p* < 0.001] and significant interactions of Task × Anterior-Posterior Axis [*F*_(2, 18)_ = 37.27, *p* < 0.001] as well as Task × Laterality [*F*_(3, 27)_ = 37.27, *p* < 0.001]. According to *post hoc* tests, in the non-attended task, the amplitudes were more negative at the frontal and central than at the posterior sites (*p* = 0.05) but there were no differences between frontal and central sites (*p* = 0.273). The amplitudes were more negative at the left and right midline electrodes: left vs. left midline (*p* = 0.005), left midline vs. right midline (*p* = 0.018), left midline vs. right (*p* = 0.021). In the attended task, the amplitudes were more negative at the frontal than at the central and posterior sites (all *p* < 0.001). The amplitudes were more negative at the left hemisphere sites: left vs. left midline (*p* = 0.001), left vs. right midline (*p* < 0.001).

In the 190–230 ms time window, there was a significant main effect of Task [*F*_(1, 9)_ = 32.94, *p* < 0.001] and significant interactions of Task × Anterior-Posterior Axis [*F*_(2, 18)_ = 9.57, *p* = 0.001], Task × Laterality [*F*_(3, 27)_ = 20.43, *p* < 0.001] as well as Task × Anterior-Posterior Axis × Laterality [*F*_(6, 54)_ = 2.85, *p* = 0.018]. According to *post hoc* tests, in the non-attended task, the amplitudes were more negative at the frontal and central than at the posterior sites (all *p* < 0.05) but there were no differences between frontal and central sites (*p* = 0.273). The amplitudes were more negative at the left and right midline electrodes: left vs. left midline (*p* = 0.002), left midline vs. right (*p* = 0.029). In the attended task, the amplitudes were more negative at the frontal than at the central and posterior sites (all *p* < 0.001). Additionally, the amplitudes were more negative at the left hemisphere sites: left vs. left midline (*p* = 0.002), left vs. right midline (*p* < 0.001). There was a significant interaction of Word Type and Anterior-Posterior Axis [*F*_(4, 36)_ = 3.12, *p* = 0.026], stemming from distributional differences between inflected and other word types; however, *post hoc* tests did not show significant differences between word types at different electrode sites.

To ensure that there were no differences between the conditions before the critical point (baseline time window), we also analyzed the responses statistically also in the pre-critical point time window (300–500 ms after the stimulus onset). The main effects of Task and Word Type were non-significant [(*F*_(1, 9)_ = 1.32, *p* = 0.296) and *F* < 1, respectively] and there were no significant interactions.

### Meg results

**Figure 4:**
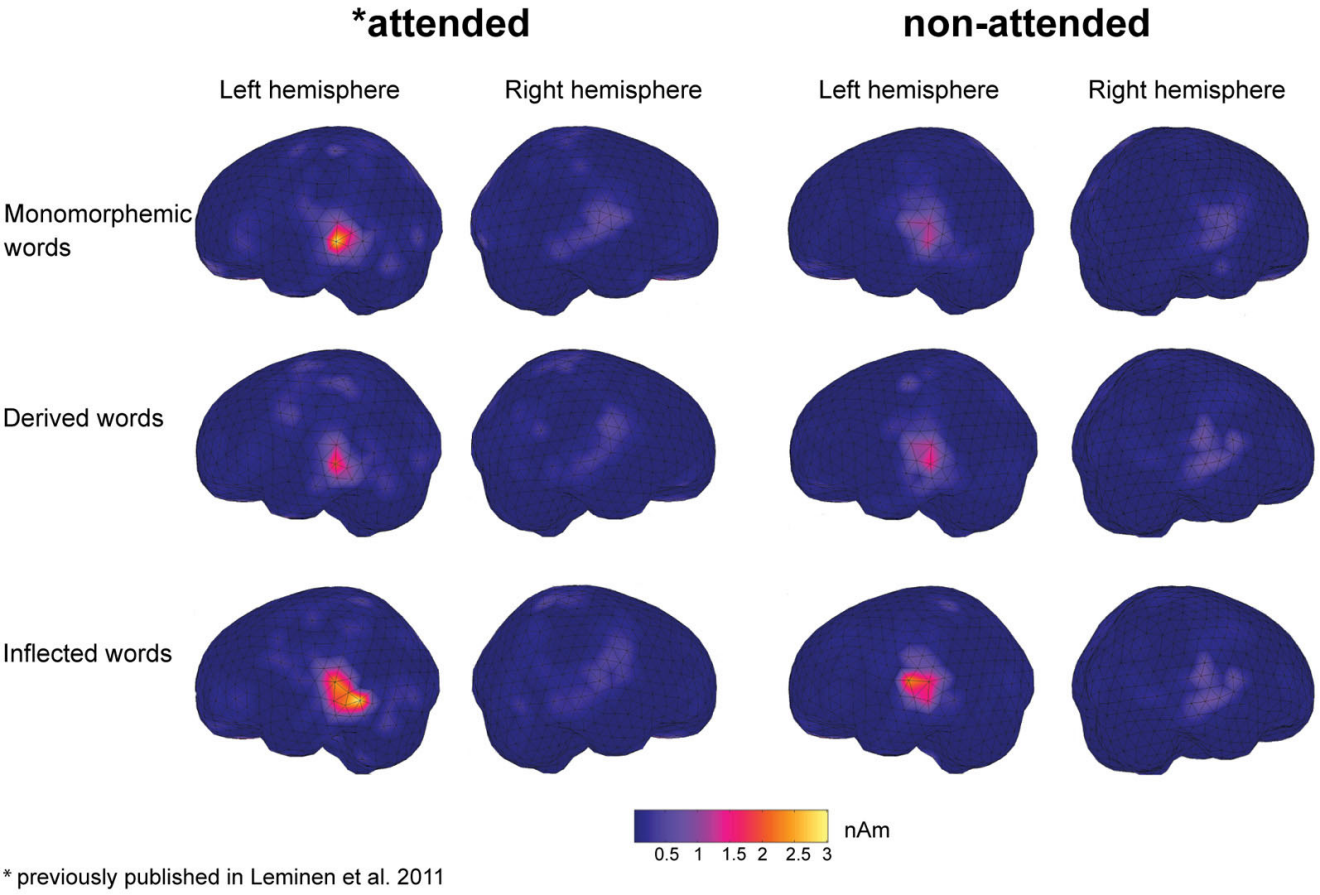
**Grand average minimum current estimates (MCE) (10 participants) calculated for monomorphemic, derived, and inflected words in the 170–210 ms time window after the critical point in the attended and non-attended tasks**.

**Figure 5:**
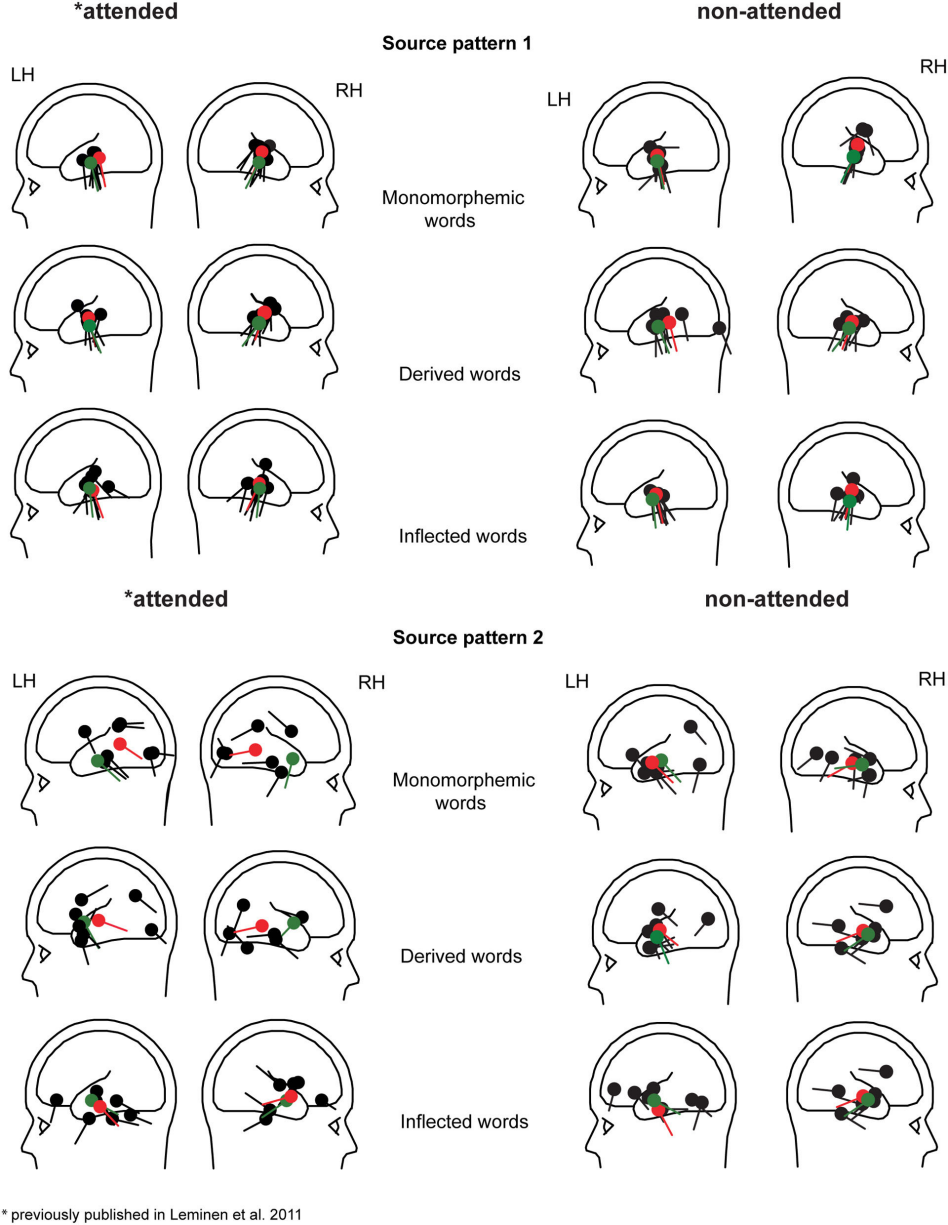
**Individual dipole locations (black) for Source patterns 1 and 2 in the left and right hemisphere for monomorphemic, derived, and inflected words in the attended and non-attended tasks**. Mean source locations (from individual dipole location, *N* = 8 in Source pattern 1 and *N* = 7 and 6 in the left and right hemispheres in Source pattern 2, respectively) and grand average (10 participants) locations are displayed as red and green dipoles, respectively.

**Figure 6:**
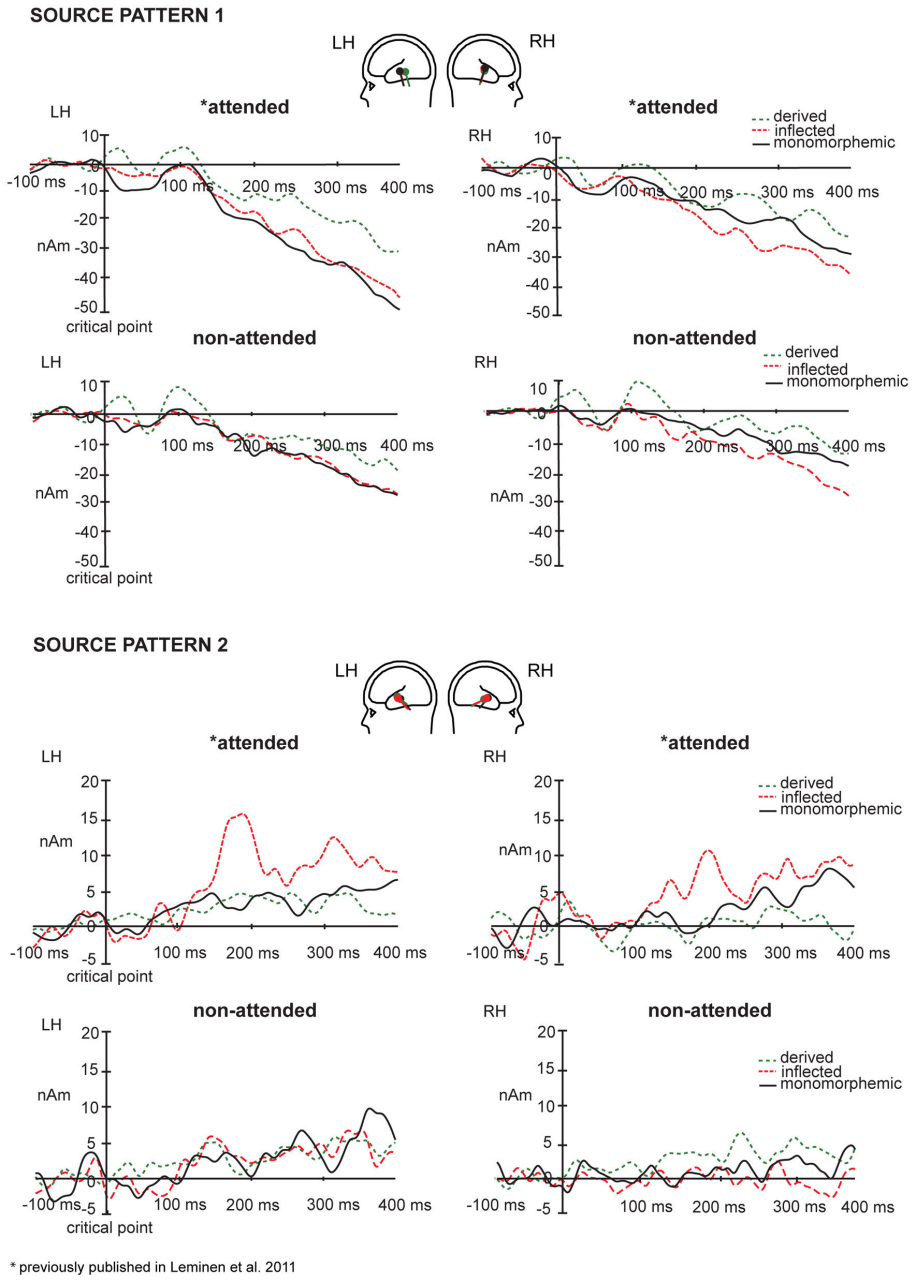
**Mean source waveforms for Source patterns 1 (above) and 2 (below) in the left and right hemisphere for monomorphemic, derived, and inflected words in the attended and non-attended tasks with baseline correction in the −100 to 0 ms window before the critical point**. Mean dipole locations for inflected, derived, and monomorphemic words in the left and right hemispheres are depicted as red, green, and black dipoles, respectively.

In grand average MCE, the non-attended processing of inflected, derived and monomorphemic words activated the superior temporal cortices less than in the attended task (Figure 4). Figure 5 displays individual source locations (black dipoles), mean (calculated from individual dipole locations) dipole locations (red dipoles), and locations estimated to the grand average data (10 subjects) (green dipoles) for Source patterns 1 and 2 in the attended and non-attended tasks. The ECDs agree with activity detected by MCE and confirm the dominance of the superior/middle temporal cortices even in the non-attended task. Figure 6 illustrates the mean source waveforms for all stimulus conditions for Source patterns 1 and 2 in the attended and non-attended tasks.

### Equivalent current dipole strengths

For the Source pattern 1, in the 80–120 ms time window after the critical point, the main effect of Task was non-significant. The main effect of Word Type was significant in both left and right hemispheres [(*F*_(2, 14)_ = 13, *p* = 0.005); (*F*_(2, 14) = 8.74_, *p* = 0.003), respectively]. *Post hoc* tests showed that in the left and right hemisphere, the source amplitudes were larger for the derived than for the inflected words (*p* = 0.002 and *p* = 0.010, respectively), and for derived than monomorphemic (*p* = 0.005 and *p* = 0.011, respectively) but there were no differences between monomorphemic and inflected words (*p* = 0.571 and *p* = 0.879). The Task × Word Type interaction was non-significant (*F* < 1).

For the Source pattern 2, in the 170–210 ms time window, Task interacted significantly with Word Type in both left and right hemispheres [*F*_(2, 12)_ = 4.65, *p* = 0.032; *F*_(2, 10)_ = 5.73, *p* = 0.022, respectively]. *Post hoc* tests did not reveal significant differences in the non-attended task, in the attended task the significant differences were observed only between inflected and derived words (larger for inflected; *p* = 0.038), (monomorphemic vs. derived: *p* = 0.810; monomorphemic vs. inflected: *p* = 0.104). Furthermore, in the 190–230 ms time window, Task interacted significantly with Word type in both left and right hemispheres [*F*_(2, 12)_ = 5.62, *p* = 0.019; *F*_(2, 10)_ = 5.43, *p* = 0.025, respectively]. *Post hoc* tests showed significant differences between the word types only in the attended task and only in the left hemisphere: inflected words showed larger source amplitudes than derived words (*p* = 0.034) and marginally significantly larger than monomorphemic words (*p* = 0.087).

## Discussion

The present study investigated the effects of attentional modulation on processing of spoken morphologically complex words. The aim was to examine whether the recently reported differences between spoken inflected and derived words (Leminen et al., 2011) take place even when the participants' attention is directed elsewhere. We assumed that the effects that are independent of attention reflect automatic activation of lexical and morpheme representations. Thus, potential differences between word types observed in the non-attended task could inform us on the nature of such representations. Here it was crucial to analyze the data by time-locking the comparisons to the onset of critical information, the suffix onset (or UP in the case of monomorphemic words). The current results show that the processing of spoken inflected vs. derived words differs not only when attention is directed to the stimuli but, importantly, also when attention is shifted to another task. Specifically, MEG results showed that the early ERPs/ERFs time-locked to the critical point (<150 ms after it) were not modulated by attention, whereas the later (>150 ms) effects were observed only in the attended task.

### The stimulus onset time-locked responses

ERPs showed an N400-like negativity for all word types both in the attended and in the non-attended task, but it was weaker in the non-attended task. Furthermore, in the attended task, the negativity was larger for inflected than derived words or monomorphemic words (Leminen et al., 2011). In the non-attended task, however, this inflectional processing cost was not observed, as there the negativity between inflected and monomorphemic words was of a similar magnitude. This finding suggests that the inflectional processing cost previously observed in several studies (e.g., Lehtonen and Laine, 2003; Lehtonen et al., 2007; Leinonen et al., 2009) and in the attended condition reflects processes dependent on attention. However, in the stimulus onset time-locked responses the time points during which particular (e.g., morphological) information becomes available cannot be exactly matched across word types, and differences observed may be due to different onset latencies at which the critical information becomes available. Therefore, firmer conclusions about morphological processing in the auditory modality can be made on the basis of the critical point time-locked responses.

### The critical point time-locked responses

ERPs showed significant early differences between the word types, more specifically in the 80–120 ms time window after the critical point. However, in that time window no interaction of word type with task was observed, indicating that the effects observed reflect processes present both in the attended and non-attended tasks. There, derived words elicited a larger negativity than inflected and monomorphemic words, whereas no differences between monomorphemic vs. inflected words were observed. It should be emphasized that these differences are not due to acoustic differences between the different suffixes (see Section “Methods” and Leminen et al., 2011). In line with the ERP results, the MEG sources at 80–120 ms after the critical point were similar in the non-attended and attended tasks: stronger source amplitudes in responses to derived than inflected or monomorphemic words. The fact that the MEG source results correspond well with the ERP findings suggests that the source of the larger negativity resides in the superior temporal cortices. To summarize, larger responses for derived than inflected words were found in this time window irrespective of the attentional situation. Thus, they appear to reflect automatic processing, assumedly related to activation of existing representations. Activation after the suffix onset elicited by the derived words thus seems to continue longer than that elicited by the inflected words. This can be explained by assuming full-form representations for derived but not inflected words.

The critical point time-locked ERPs also showed that no significant differences between word types were elicited in the non-attended task in any of the measures at later stages after the critical point. In contrast, when only the attended data was analyzed (Leminen et al., 2011), in the 190–230 ms time window, inflected words elicited a larger left-lateralized negativity than both monomorphemic and derived words. This negativity was particularly prominent at fronto-temporal electrode sites (Leminen et al., 2011). In MEG, only in the attended task, inflected words elicited larger source amplitudes at temporal cortical areas than derived words in the left hemisphere in the 190–230 ms time window. This suggests that the left-lateralized negativity is generated in the left temporal cortices. Following accounts on left anterior negativity effects in processing inflected words (Penke et al., 1997; Rodríguez-Fornells et al., 2001; Morris and Holcomb, 2005), the left-lateralized negativity and left temporal source activity in the attended task might reflect building a morphosyntactic context for construction of meaning of the morpheme combination (Leminen et al., 2011). However, when participants' focus is directed to another modality, this assumedly combinatorial activity is drastically diminished, suggesting that this process requires initiation of conscious attention.

The activation not dependent on attention, i.e., one observed in the non-attended and attended tasks alike, is likely to reflect automatic access of the auditory input to existing word representations. Hearing a word involves activating the stored representations of the existing cohort candidates (Marslen-Wilson, 1987), and the continued mapping after the critical point indicates existing representations that match the input of the full-forms. For derived words, the activation continued after the suffix onset, shown by a difference to the other word types at 80–120 ms after the suffix onset^4^. In contrast, for inflected words, the activation halted after the stem had been heard, suggesting that no full-form representation exists for it. For monomorphemic words, the critical point was the UP. Thus, the matching lexical representation for such words was already activated at the critical point and there was nothing else in the cohort to be automatically activated, indexed by the smaller negativity and source amplitudes for them than for derived words.

These results of morphological processing without attending to the stimuli are in line with previous ERP/MEG findings recorded in paradigms requiring focused attention (Lehtonen et al., 2007; Leinonen et al., 2009; Vartiainen et al., 2009; Leminen et al., 2011). The findings support the view that most Finnish inflected words do not have full-form representations (with a possible exception of very high frequency inflected forms, see e.g., Soveri et al. (2007) and Lehtonen and Laine, 2003), but derived words do. The interpretation of full-form representations for derived words is in accordance with the results from the present attended task (Leminen et al., 2011) and with several other previous behavioral (Niemi et al., 1994; Bertram et al., 1999; Vannest et al., 2002; Järvikivi et al., 2006), eye-tracking (Hyönä et al., 1995), and neuroimaging studies (Bozic et al., 2009). In the attended task, the stimulus onset and critical point time-locked ERPs and MEG sources in the later time windows (>150 ms after the critical point) elicited by derived and monomorphemic words did not differ (Leminen et al., 2011). Behavioral and eye-tracking studies in Finnish have typically shown equal reaction times, error rates, and fixation durations for the derived and monomorphemic words (Niemi et al., 1994; Hyönä et al., 1995; Bertram et al., 1999; Vannest et al., 2002). This has been taken as evidence for the existence of full-form representations for derived words. Recent fMRI findings demonstrated that derivational affixes in English do not selectively activate left-lateralized fronto-temporal areas as do inflected words, suggesting that derivational affixes may not trigger decompositional processes in the same way as inflectional affixes (Bozic et al., 2009).

The early activation after the critical point in the non-attended task was also elicited in the attended task (Leminen et al., 2011). Since this stage of word recognition was not modulated by attention, it may reflect a rather automatic process, the activation of stored morpheme or full-form representations. In contrast, the later effects seen in the attended task were not observed in the non-attended task: even though the Task by Word Type interaction did not reach significance in the ERP analysis, such an interaction was observed in the MEG data. In the attended condition (Leminen et al., 2011), inflected words elicited stronger or more negative responses than monomorphemic words in the 190–230 ms time window (albeit only marginally significantly in the source amplitudes, but nevertheless showing a similar trend as in ERPs). Thus, the inflectional processing cost, i.e., larger effects for inflected than monomorphemic words, was observed only later in time (at ~200 ms after the critical point) and was modulated by attention. In line with earlier views based on results in attended tasks such as lexical decision or acceptability judgments (e.g., Lehtonen et al., 2007; Leminen et al., 2011), this processing cost can thus be interpreted to stem from a later, semantic-syntactic stage of decomposition and reflect a controlled integration process.

On a more general level, the results from these two studies suggest an early automatic mapping of incoming acoustic information to stored representations and a later, more active compositional process at a (morpho)syntactic-semantic level, which does not take place without attention to the stimuli. In line with a recent review (Shtyrov, 2010), the earliest stages of linguistic responses (<150 ms) appear to be immune to attentional demands and may thus be automatic, whereas attention-modulated effects on both lexical and syntactic ERPs emerge later (>150 ms). Language automaticity is thus limited to the very first stages of linguistic processing, with respect to the point in time where the relevant information is available in the auditory input, such as suffix onset and UP in the current study. This early automaticity may be explained by robustness of strongly connected linguistic memory circuits in the brain that can activate fully even when attentional resources are low (Shtyrov, 2010). Later stages of speech analysis, such as (morpho)syntactic licensing and/or semantic integration of morpheme combination, seem to be affected by the attentional control and may thus depend on it.

### Conflict of interest statement

The authors declare that the research was conducted in the absence of any commercial or financial relationships that could be construed as a potential conflict of interest.

## Footnotes

^1^Due to the limited amount of available derived stimuli, we were not able to match fully all the conditions with regard to frequency. While the derived and inflected word groups were well matched for log surface frequency (*p* = 1.000), the monomorphemic words were more frequent than inflected and derived words (*p* < 0.05). Yet, this difference is unlikely to be the source of the observed effects: it is likely that the higher frequency would only have decreased the observed differences, and assumedly in the same way for derived and inflected words. The log base frequency values differed across conditions (all *p*-values < 0.05). To exclude the possibility that the differences in the ERP responses could be due to frequency differences, we conducted separate ANOVA analyses 300–500 ms after the stimulus onset and −200 to 0 ms before the critical point, the time windows when stem access took place. None of these analyses showed significant effects within each critical time-window (for a similar approach, see Janssen et al., 2006). The results of these tests thus clearly show that there were no significant differences in the ERP amplitude during the base morpheme access, and that the possible base frequency differences did not have notable effects on the ERP responses.

^2^When comparing the words presented in the non-attended vs. the attended situation, there was no main effect of Task or Task × Word Type interaction in stimulus properties (base and surface frequency, F0, intensity, suffix/UP onset and stimulus duration), all *F* < 1.

^3^Leminen et al. (2011) only report results for the attended situation whereas the present ANOVAs include Task (attended, non-attended) as a factor. Consequently, the analyses reported are not exactly the same as those in Leminen et al. (2011).

^4^Solomyak and Marantz (2010) have observed that stem-affix transitional probability modulates early responses during visual word recognition. As this variable was not controlled in the present study, a possible transitional probability effect cannot be excluded, and future studies should focus on this variable also in the auditory domain.
